# Infodemic, Institutional Trust, and COVID-19 Vaccine Hesitancy: A Cross-National Survey

**DOI:** 10.3390/ijerph19138033

**Published:** 2022-06-30

**Authors:** Xi Chen, Woohyung Lee, Fen Lin

**Affiliations:** 1 Department of Sociology and Social Policy, Lingnan University, Hong Kong, China; chenxi424@gmail.com; 2 The JC School of Public Health and Primary Care, The Chinese University of Hong Kong, Hong Kong, China; woohyunglee@cuhk.edu.hk; 3 Department of Media and Communication, City University of Hong Kong, Hong Kong, China

**Keywords:** infodemic, information overload, misinformation, vaccine hesitancy, institutional trust, COVID-19

## Abstract

The COVID-19 pandemic has been accompanied by a massive infodemic. Yet limited studies have quantified the impact of the COVID-19 infodemic on vaccine hesitancy. This study examined the effect of perceived information overload (IO) and misinformation on vaccine willingness and uptake within a cross-national context. It also investigated how trust in multiple institutions affected vaccine outcomes and moderated the relationship between the infodemic and vaccine attitude and behavior. A cross-national online survey of residents, representative of the general population aged ≥18 in six Asian and Western jurisdictions, was conducted in June 2021. The results showed that perceived IO was positively associated with COVID-19 vaccine willingness and uptake. Belief in misinformation was negatively associated with vaccine willingness and uptake. Institutional trust may increase vaccine willingness and uptake. Moreover, trust in the government and civil societies tended to strengthen the positive effect of IO and reduce the negative impact of misinformation on vaccine willingness and uptake. The relationship between belief in misinformation and getting vaccinated against COVID-19 was unexpectedly stronger among those with a higher level of trust in healthcare professionals. This study contributes to a better understanding of the main and interactive effect of the infodemic and institutional trust on vaccine outcomes during a pandemic.

## 1. Introduction

As of 11 February 2022, there have been more than 404.9 million confirmed cases of COVID-19 and over 5.8 million related deaths [1]. Achieving high coverage of COVID-19 vaccination is critical for containing the pandemic and protecting the community. However, vaccine hesitancy, which refers to “[a] delayed acceptance or refusal of the vaccine despite the availability of the vaccination services [2]”, is prevalent among COVID-19 vaccine skeptics. An analysis of US households from January to March 2021 showed that 18.4% of participants reported that they would probably or definitely not get a COVID-19 vaccine [3]. The UK Household Longitudinal Study in late 2020 found an overall COVID-19 vaccine hesitancy rate of 18% [4]. A global survey in June 2020 showed that about 20.2% of respondents in South Korea and 32.1% in Singapore were hesitant to receive COVID-19 vaccines [5]. A survey of Hong Kong residents in April 2020 showed that 25.5% had no intention of receiving the COVID-19 vaccines [6]. A survey of Japanese people in January 2021 showed a rate of vaccine hesitancy of 37.9% [7].

Hesitancy regarding the COVID-19 vaccine may be rooted in the “infodemic”, which is defined by the World Health Organization (WHO) as “an overabundance of information—some accurate and some not—that makes it hard for people to find trustworthy sources and reliable guidance when they need it” [8]. Anti-vaccination misinformation may induce fear and undermine trust in health institutions and programs among citizens, thereby increasing their vaccine hesitancy and reducing vaccine uptake. A randomized controlled trial in the UK and US showed that exposure to online misinformation regarding COVID-19 vaccines, relative to factual information, reduced 6.2–6.4 percentage points in vaccination intent among those who initially stated that they would definitely accept a vaccine [9]. However, limited studies have quantified the impact of misinformation on individuals’ vaccination decisions beyond the Western context.

Besides, the infodemic is likely to cause a feeling of information overload (IO) when the amount of information to which people are exposed to exceeds the optimal level that they can process and understand effectively [10]. As COVID-19 vaccines are developed quickly, information about their quality, safety, and effectiveness involves a high level of uncertainty and complexity, which may contribute to a perception of IO, as processing COVID-19 information requires great cognitive resources. Prior research has revealed that health IO may lower the willingness to use medical services [11], hinder the dissemination of health knowledge, and reduce health behaviors [11,12]. However, evidence regarding the impact of IO on compliance behavior during the COVID-19 pandemic is mixed [13,14]. To our best knowledge, no research has examined the effect of perceived IO on vaccine hesitancy related to COVID-19.

In addition to considering how the infodemic may affect vaccine hesitancy, an important contribution of our paper is to assess the role of institutional trust. Institutional trust refers to the trust that people place in a system or an institution such as a government, a political party, a non-governmental organization (NGO), or a particular public or private organization. Vaccine confidence is believed to stem from the trust in the institutions and actors that develop and administer the vaccination programs [15]. Extant studies have revealed that trust in the government can reduce vaccine hesitancy regarding COVID-19, probably because people who trust in the government have more confidence in the effectiveness and safety of vaccines, as well as higher faith in the government’s ability to procure and deliver vaccines efficiently and fairly [16]. Similarly, trust in key stakeholders such as healthcare professionals and NGOs may increase the trust in the scientific evidence regarding the safety, effectiveness, and importance of COVID-19 vaccines, thus serving as a crucial underpinning for individual decision-making on vaccination [17]. While vaccine-related trust exists within the political, medical, and social sectors, few studies have examined how different types of trust may moderate the effect of the COVID-19 infodemic and influence vaccination.

Despite emerging studies addressing the pressing issue of the COVID-19 infodemic, most of these studies were from a computational or exploratory perspective [18,19]. In this work, we employed a survey-based approach to quantify the impact of the COVID-19 infodemic on vaccination intention and behavior. According to the definition of an infodemic, we conceptualized the infodemic as a perceived information overload and belief in misinformation. Using data obtained from six Asian and Western societies in June 2021, this study complements existing studies on the infodemic and vaccine hesitancy during the COVID-19 pandemic by addressing two aspects. First, it was among the first attempt to quantify the impact of vaccine-related IO and misinformation consumption on the willingness to receive COVID-19 vaccines, and actual vaccination within a cross-national context. Second, it investigated how trust in multiple institutions affected vaccination and moderated the relationship between exposure to the infodemic and vaccine attitudes and uptake. The findings of this research may add to our understanding of the main and interactive effects between the infodemic and institutional trust on vaccine willingness and uptake. Understanding how the infodemic differentially impacts individuals with different levels of institutional trust can also motivate the design of relevant interventions to reduce the potential hazard of an infodemic.

## 2. Materials and Methods

### 2.1. Study Design and Data Collection

The data came from a cross-national online survey of public attitudes and responses toward COVID-19 in six jurisdictions in Asian and Western societies, including Hong Kong, Japan, South Korea, Singapore, the UK, and the US. The COVID-19 vaccines are free and available for residents in these six societies. The survey was conducted between 15 June and 30 June 2021. Reporting adheres to the checklist for reporting results of internet e-surveys (CHERRIES) [20]. The methods were provided here in brief; additional details can be found in Supplementary SI.

The surveys were completed using the online panels provided by a global research agency, which helped us obtain a probability-based sample of the required size and facilitate quick completion for time-sensitive projects [21], particularly during the COVID-19 pandemic. The survey targeted residents aged ≥ 18. A quota sampling strategy was adopted to ensure that the samples chosen matched the population’s geographical and demographic characteristics released by the latest available census in each jurisdiction. The usability and technical functionality of the electronic questionnaire had been tested by the research team during the pilot stage of this study. A total number of 6764 representative responses were collected, with approximately 1100 individuals in each jurisdiction. The questionnaire was available in English, Chinese, Korean, and Japanese for participants from different jurisdictions. The study was approved by the Human Subject Ethics Committee of the [omitted due to anonymity]. Participant consent has been obtained. The project team guaranteed that all the information would be treated with complete confidentiality and anonymity.

### 2.2. Measures

Vaccine hesitancy was assessed by both acceptance and uptake of COVID-19 vaccines. Vaccine acceptance was gauged by the question: “A few vaccines have been developed to prevent coronavirus; would you accept it for yourself? (1 = definitely not; 7 = definitely yes). Vaccine uptake was a binary variable of whether the participants had received the COVID-19 vaccine (at least one dose) at the time of the survey (yes/no).

Exposure to the COVID-19 infodemic was assessed by belief in misinformation and perceived IO regarding COVID-19 vaccines. Belief in misinformation was gauged by asking the participants whether they agreed with three widespread false claims about the COVID-19 vaccines [21]. The measure of perceived information overload was adapted from Farooq et al. (2020), which included two items: “I receive too much information regarding the COVID-19 vaccine to form a coherent picture of what has been happening in the past four weeks;” and “I am often distracted by the excessive amount of information and news from multiple sources about the COVID-19 vaccine [22].

Institutional trust was gauged by asking the respondents to rate on a 7-point Likert scale the level of trust (1 = totally do not trust; 7 = totally trust) toward the government, healthcare professionals, and NGOs, with higher scores indicating higher levels of trust in each institution.

Detailed information on survey questions and coding methods are shown in Supplementary SII.

### 2.3. Data Handling and Analysis

We used descriptive statistics to calculate frequencies of prevalence in numbers and their percentages. For normally distributed continuous data, we calculated means and their standard deviations, and for non-normally distributed data, we calculated medians and their interquartile ranges. We used ordinary least squares (OLS) or logistic regression models to examine the associations between various background variables and two outcome variables, vaccine hesitancy (continuous) and vaccine uptake (binary). We then examined the effects of COVID-19 vaccine-related IO and misinformation on the two outcome variables after adjusting for various sociodemographic backgrounds. Lastly, two-way interaction terms were computed between the three types of institutional trust and perceived IO/belief in misinformation about COVID-19 vaccines. All the analyses were performed using Stata 16.0. Unstandardized coefficients with 95% confidence intervals were reported. Statistical significance was set as *p* < 0.05.

## 3. Results

A total of 6764 responses were received during the study period. Responses with incomplete information were deleted (*n* = 571), and therefore the final sample size was 6193. We also used multiple imputation methods to impute the missing observations and carried out the same analysis. The results were not significantly different from the sample using the complete responses. We thus reported the results using the complete information.

Table 1 displays the background characteristics of the respondents. In the full sample, about one-fifth were aged between 18 and 29 (21.8%), or 60 or above (23.0%), and more than half (55.3%) were aged between 30 and 59. There were slightly more females (50.1%) than males (49.9%). More than 40% of the respondents had a college education or above (45.6%). A majority of respondents were professional or service workers (65.1%), lived in urban areas (79.5%), and without chronic diseases (81.6%). As for COVID-19 infection status, about 5.2% of respondents reported being infected with COVID-19, and 8.4% reported that their family members had been infected with COVID-19. The background characteristics of participants in each jurisdiction were presented in the Supplementary (Table S1). The infection rate of COVID-19 among the respondents and their family members was remarkably higher in the UK (10.3% and 17.9%) and the US (13.9% and 22.8%), compared to respondents in Asian societies, where the infection rate ranged from 0.7 to 2.2% among respondents and their family members.

Background factors influencing the decision to take a COVID-19 vaccine are presented in Table 2. The results showed that older (versus respondents aged 18–29) and male respondents were more willing to accept COVID-19 vaccines and were more likely to be vaccinated against COVID-19. Respondents with higher socioeconomic status, indexed by higher education, higher income quantiles, and urban residence, were more willing to accept COVID-19 vaccines and more likely to get at least one dose of the vaccine. In addition, having chronic disease was positively associated with COVID-19 vaccine willingness and uptake. The COVID-19 infection among the respondents and their family members had a positive effect on the uptake of COVID-19 vaccines but no significant effect on their willingness to get vaccinated. Participants may not necessarily change their perception of COVID-19 vaccines or their willingness to accept vaccines after being infected, but they may be motivated to take COVID-19 vaccines so as to protect against a new infection, mitigate symptoms, and protect others.

Table 3 shows the main and interactive effects of the infodemic and institutional trust on vaccine acceptance and uptake, adjusting for background factors. Model 1a shows that perceived IO was positively associated with acceptance of COVID-19 vaccines (b = 0.20, 95% CI = [0.16, 0.23], *p* < 0.001), while belief in misinformation was negatively associated with vaccine acceptance (b = −0.31, 95% CI = [−0.34, −0.29], *p* < 0.001). Similarly, regarding the uptake of COVID-19 vaccines (Model 1b), perceived IO had a positive effect (Model 1b; b = 0.13, 95% CI = [0.08, 0.18], *p* < 0.001) and belief in vaccine misinformation had a negative effect (b = −0.20, 95% CI = [−0.25, −0.15], *p* < 0.001) on vaccine uptake.

Models 2a and 2b examine the effect of institutional trust on vaccine willingness and uptake. The results in Model 2a confirmed that trust in the government (b = 0.25, 95% CI = [0.22, 0.28], *p* < 0.001) and healthcare professionals (b = 0.27, 95% CI = [0.23, 0.32], *p* < 0.001) were positively associated with COVID-19 vaccine acceptance. The results were similar when it came to vaccine uptake (Model 2b). Higher trust in the government (b = 0.18, 95% CI = [0.12, 0.24], *p* < 0.001) and healthcare professionals (b = 0.16, 95% CI = [0.09, 0.24], *p* < 0.001) were associated with a higher likelihood of getting vaccinated. However, trust in NGOs was not significantly related to COVID-19 vaccine acceptance and behavior.

**Figure 1:**
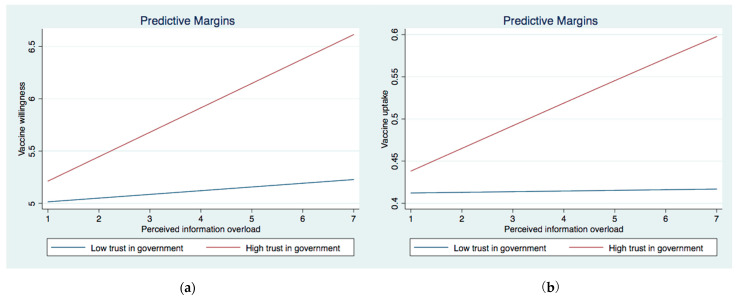
(**a**) Interaction between perceived information overload and trust in the government trust on vaccine willingness. (**b**) Interaction between perceived information overload and trust in the government trust on vaccine uptake.

**Figure 2:**
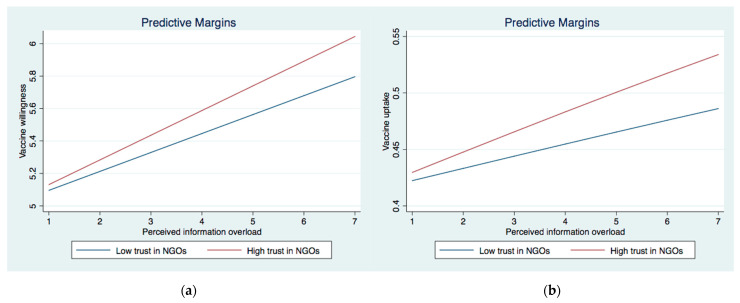
(**a**) Interaction between perceived information overload and trust in NGOs on vaccinewillingness. (**b**) Interaction between perceived information overload and trust in NGOs trust on vaccine uptake.

**Figure 3:**
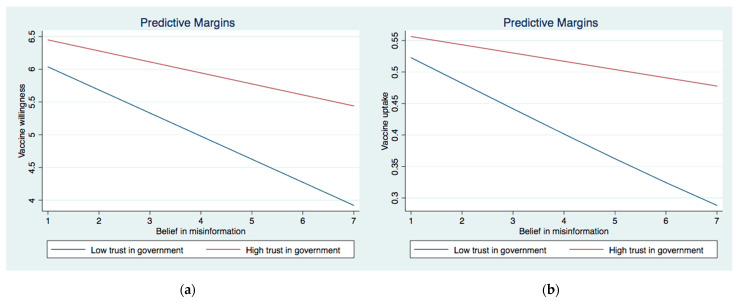
(**a**) Interaction between belief in misinformation and trust in the government on vaccinewillingness. (**b**) Interaction between belief in misinformation and trust in the government on vaccine uptake.

**Figure 4:**
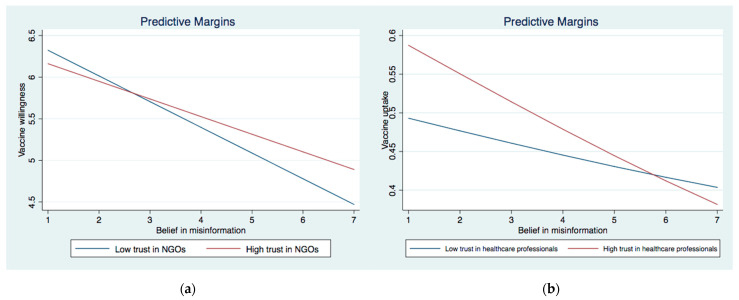
(**a**) Interaction between belief in misinformation and trust in NGOs on vaccine acceptance. (**b**) Interaction between belief in misinformation and trust in healthcare professionals on vaccine uptake.

We then investigated the interactive effect between institutional trust and perceived IO (Models 3a and 4a) and belief in misinformation (Models 3b and 4b) on vaccine willingness and uptake. In Model 3a, the interaction between perceived IO and trust in the government as well as the interaction between perceived IO and trust in NGOs were significant. The significant interaction effects were graphically demonstrated in Figure 1a and Figure 2a. They showed that the association between perceived IO and COVID-19 vaccination acceptance was stronger among those with higher levels of trust in the government and NGOs than their counterparts who showed lower levels of trust in those two institutions. As for the interaction between belief in misinformation and institutional trust, two significant interaction terms emerged for COVID-19 vaccine acceptance (Model 4a), i.e., MI × trust in the government and MI × trust in NGOs. These significant interactive effects were graphically demonstrated in Figure 3a and Figure 4a. The results showed that the negative effect of belief in misinformation on vaccine acceptance was stronger among those with lower levels of trust in the government and NGOs than those with higher levels of trust in such institutions.

**Figure 5:**
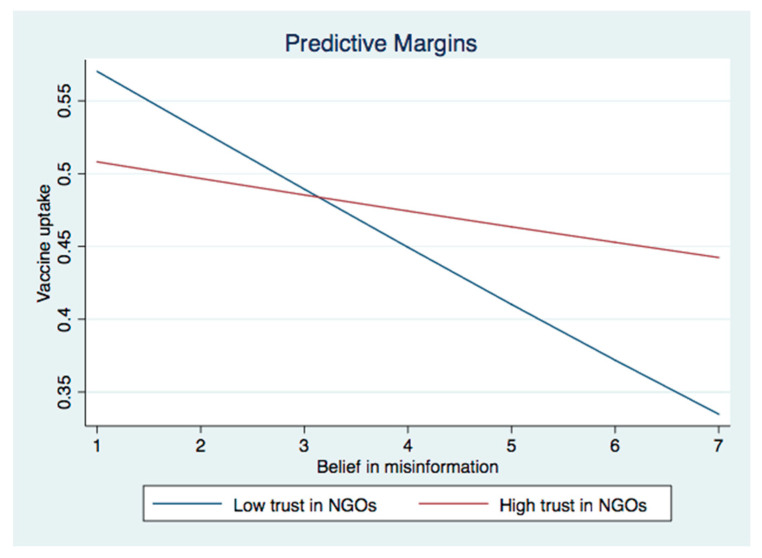
Interaction between belief in misinformation and trust in NGOs on vaccine uptake.

The results were largely similar when it came to vaccine uptake. The relationship between perceived IO and vaccine uptake was strengthened among those with higher levels of trust in the government and NGOs, as illustrated in Figure 1b and Figure 2b. In addition, the association between the belief in misinformation and vaccine uptake was stronger among those with lower levels of trust in the government (Figure 3b) and NGOs (Figure 5). Unexpectedly, the relationship between belief in misinformation and getting vaccinated against COVID-19 was stronger among those with a higher level of trust in healthcare professionals (Figure 4b). In other words, respondents having higher trust in healthcare professionals were more vulnerable to COVID-19 vaccine-related misinformation and were less likely to be vaccinated.

## 4. Discussion

This study was among the first to investigate the effect of exposure to the infodemic on vaccine hesitancy and explore the moderating role of institutional trust. The data were obtained from a large-scale cross-sectional survey in six Asian and Western societies in June 2021. Our results revealed a positive effect of perceived IO and a negative effect of belief in misinformation on COVID-19 vaccine acceptance and uptake. Trust in the government and healthcare professionals tended to increase the willingness and uptake of COVID-19 vaccines. Besides, institutional trust may moderate the effect of the infodemic on vaccine acceptance and uptake. Trust in the government and NGOs may strengthen the effect of perceived IO and reduce the effect of belief in misinformation on vaccine acceptance and uptake. Nevertheless, the association between belief in misinformation and vaccine uptake was stronger among people with higher levels of trust in healthcare professionals. The findings of this study contribute to a better understanding of the main and interactive effect between the infodemic and institutional trust on vaccine outcomes during an unprecedented pandemic. We discussed the main findings below.

First, our results showed a high level of exposure to the infodemic regarding COVID-19 vaccines. About 41.5% and 46.1% of respondents reported that they received too much information about COVID-19 vaccines and were often distracted by such information to form a coherent picture (refer to Table S3 in the Supplementary). In addition, more than one in three respondents thought it is true that “the real purpose of a mass vaccination program against COVID-19 is to track and control the population” (36%), “the COVID-19 vaccine will alter human DNA” (33.1%), and “the only reason the COVID-19 vaccine is being developed is to make money for pharmaceutical companies” (33.8%) (refer to Table S3 in the Supplementary). Such prevalence rates were considerably higher compared to a survey conducted in November 2020 in the UK, showing the percentages of believing the above statements to be 14%, 9%, and 15%, respectively [21]. Despite accumulating evidence of vaccine safety and effectiveness, misinformation about COVID-19 vaccines does not necessarily diminish but increases significantly [23]. Such an alarming finding calls for more research and intervention to reduce COVID-19 vaccine-related misinformation.

Second, while belief in vaccine misinformation tended to decrease the willingness to vaccinate and vaccine uptake, receiving excessive information about COVID-19 vaccines may increase vaccine intent and uptake. The adverse impact of exposure to COVID-19 misinformation on vaccine intent has been well documented in prior studies [9]. However, the positive relationship between perceived IO and vaccine uptake shown in our results seemed inconsistent with prior research on health IO [13,14]. This is perhaps because more exposure to COVID-19-related information may cause intense worry about infection and increase protective behavior as a result [16]. These findings, however, do not imply that IO is entirely beneficial. Studies have confirmed that an overabundance of COVID-19 information can harm mental wellbeing and lead to a discontinuation of information seeking as people deliberately avoid information that threatens their wellbeing [24]. Perceived IO is thus detrimental to public health as the acquisition of health information helps individuals make informed medical decisions and engage in preventive behavior.

Third, our study underlined the role of institutional trust in promoting vaccine coverage. Vaccine-related trust is a multifaceted construct, and we found that trust in one’s government and healthcare professionals in handling the pandemic may increase the willingness to vaccinate and the uptake of COVID-19 vaccines. Such findings were consistent with prior research showing that during a disease outbreak, trust in governments, scientists, and civil citizens are the premise for behavioral change [25]. Restoring and increasing institutional trust will be crucial to achieving herd immunity vaccination levels. However, sustaining trust can be challenging in times of uncertainty and risk, given that institutional trust tends to decrease during a crisis, including the current COVID-19 pandemic [26].

Moreover, we provided new evidence on how institutional trust may moderate the effect of the infodemic on individuals’ vaccine willingness and uptake. People who placed higher trust in the government and civil society were more resilient to vaccine misinformation and less likely to reduce their vaccine willingness and uptake. It suggested that governments and community organizations played a critical role in dispelling misinformation and instilling greater empowerment within the audience of misinformation to heighten their resistance to its pernicious effects on vaccination. Unexpectedly, although trust in healthcare professionals may increase the willingness to receive COVID-19 vaccines and the likelihood of getting vaccinated, the relationship between the belief in misinformation and vaccine uptake was stronger among those having more trust in the public health systems. Despite higher workplace exposure and contact with potentially at-risk patients, COVID-19 vaccine hesitancy was prevalent among healthcare workers, partly due to scientific complexities related to the COVID-19 vaccine, particularly the antigen test platforms that are not yet familiar to healthcare professionals. Low acceptance rates were observed among Hong Kong nurses in two studies [27,28], and COVID-19 vaccine hesitancy among nurses in Japan was 1.4 times higher than that among the general population [25]. COVID-19 vaccine hesitancy also persisted among healthcare workers in the West; hesitancy may remain even after vaccine acceptance (i.e., passive acceptance) [29]. Future studies may further investigate the reasons for vaccine hesitancy among healthcare workers. Given that healthcare professionals have a powerful influence on patient vaccination decisions, people placing trust in healthcare professionals may be more vulnerable to vaccine misinformation. Besides, it might be because people who trust healthcare professionals to effectively control the pandemic had higher confidence in them to contain COVID-19, which alleviated their fear and worry. Those who believed in the vaccine misinformation may be particularly less motivated to receive COVID-19 vaccines when they trust the ability of the public health system to cure the disease and aid the controlling of the pandemic.

This study has several practical implications. First, our study found that exposure to vaccine misinformation was a risk factor for COVID-19 vaccine hesitancy, while exposure to excessive vaccine-related information may increase vaccine uptake. Therefore, to reduce vaccine reluctance during the pandemic, it is important to adopt strategies to tackle vaccine-related misinformation and improve information quality. From the information provider side, governments and media should disseminate evidence-based and transparent information swiftly and widely among the public. In addition, there is a need for public education and training to increase individuals’ information literacy and equip them with the skills to examine information credibility. Moreover, various measures should be taken to increase citizens’ institutional trust. Governments should release timely information on vaccination strategies and increase the competence and reliability of the institutions that deliver vaccination programs. There is also a need for collaboration among various sectors and stakeholder groups (e.g., government, healthcare professionals, and civil society) to combine their various strengths to enhance transparent and coherent public communication in order to enhance the public’s trust in vaccines. Notably, numerous international vaccination campaigns have led to a significant rise in vaccination rates in many societies [30] compared to the time of data collection in this study. Nevertheless, our findings revealed that the infodemic and institutional trust are at least partially responsible for early vaccine hesitancy and refusal. Moreover, false claims about the safety and efficacy of COVID-19 vaccines persist, preventing some people from getting vaccinated. In order to increase vaccination rates, it is imperative to continue tackling the infodemic.

This study has several limitations. The data were collected at a single time point of the pandemic, while the pandemic continues to evolve. We relied upon self-reports of exposure to the infodemic, rather than objective media logs. Although we conducted surveys in both Asia and the West, we only focused on well-developed societies in these regions. Longitudinal studies in both developed and under-developed countries are desirable to monitor the change in vaccination attitudes and behavior over time. Moreover, we did not specify the types of NGOs in the survey and only asked participants about their trust in local NGOs in general. Future studies may further identify the role of different types of NGOs in providing information about the pandemic and encouraging vaccination and health behaviors recommended by the WHO. Nevertheless, with nationally representative samples in six societies in the East and West at the time of the initial COVID-19 vaccine rollout, examining the core topics of misinformation, IO, and institutional trust, this study provides a unique and vital window into the COVID-19 infodemic and vaccine hesitancy.

## 5. Conclusions

This study contributes to a better understanding of the main and interactive effect of the infodemic and institutional trust on vaccine outcomes during an unprecedented pandemic. Our findings showed that perceived IO had a positive effect, and belief in vaccine misinformation exerted a negative effect on COVID-19 vaccine acceptance and uptake. Trust in the government and healthcare professionals may increase the willingness and uptake of COVID-19 vaccines. In addition, trust in the government and NGOs tended to strengthen the effect of perceived IO on vaccine acceptance and uptake, and reduce the effect of the belief in misinformation on vaccine willingness and uptake. However, the association between the belief in misinformation and vaccine uptake was stronger among people with higher levels of trust in healthcare professionals. The findings of this study contribute to a better understanding of the main and interactive effect between the infodemic and institutional trust on vaccine outcomes during an unprecedented pandemic. It is important to adopt strategies to tackle vaccine-related misinformation and increase information quality. There is also a need for collaboration among various institutions to combine their various strengths to enhance transparent and coherent public communication to build public trust in vaccines.

## Figures and Tables

**Table 1:** Sample characteristics (*n* = 6193).

Variable			Variable		
Age	*n*	%	COVID-19 infection of the respondent	*n*	%
18–29	1349	21.8	No	5828	94.1
30–59	3423	55.3	Yes	324	5.2
≥60	1421	23.0	Unknown/prefer not to answer	41	0.7
Sex			COVID-19 infection of family members		
Male	3090	49.9	No	5624	90.8
Female	3103	50.1	Yes	521	8.4
Education			Unknown/prefer not to answer	48	0.8
≤Secondary	1365	22.0	Jurisdiction		
≥Tertiary	2826	45.6	Hong Kong	1025	16.6
Unknown/prefer not to answer	2002	32.3	Japan	1032	16.7
Occupation			Singapore	1086	17.5
Professional/service worker	4033	65.1	South Korea	1084	17.5
Manual worker	561	9.1	UK	988	16.0
Other/prefer not to answer	1599	25.8	US	978	15.8
Income			Uptake of COVID-19 vaccines		
Lowest quartile	1525	24.6	Yes	3249	52.5
2nd quartile	1529	24.7	No	2944	47.5
3rd quartile	1855	29.9	Acceptance of COVID-19 vaccines, mean (SD)	5.56	(1.72)
Highest quartile	959	15.5	Perceived information overload, mean (SD)	4.18	(1.49)
Unknown/prefer not to answer	325	5.3	Belief in misinformation, median (IQR)	4	(2–5)
Area			Trust in the government, median (IQR)	5	(3.5–6)
Urban	4922	79.5	Trust in healthcare professionals, median (IQR)	5	(5–6)
Rural	1271	20.5	Trust in NGOs, median (IQR)	4	(4–5)
Chronic disease					
No	5056	81.6			
Yes	1012	16.3			
Unknown/prefer not to answer	125	2.0			

**Table 2:** Sociodemographic characteristics and COVID-19 vaccine hesitancy and vaccine uptake.

	Willingness to Accept COVID-19 Vaccines		Uptake of COVID-19 Vaccines	
	b ^†^	[95% CI]	b ^‡^	[95% CI]
Age (ref: 18–29)				
30–59	0.12 *	[0.01, 0.22]	0.44 ***	[0.29, 0.60]
≥60	0.69 ***	[0.57, 0.82]	1.41 ***	[1.21, 1.61]
Sex (ref: male)				
Female	−0.15 ***	[−0.23, −0.06]	−0.37 ***	[−0.49, −0.24]
Education (ref: ≤secondary)				
≥Tertiary	0.25 ***	[0.14, 0.36]	0.26 **	[0.09, 0.44]
Unknown	0.21 ***	[0.09, 0.34]	0.41 ***	[0.23, 0.60]
Occupation (ref: professional or service worker)				
Manual worker	−0.07	[−0.22, 0.08]	−0.05	[−0.27, 0.18]
Other	−0.01	[−0.12, 0.09]	−0.09	[−0.25, 0.08]
Income (ref: lowest quartile)				
2nd quartile	0.24 ***	[0.13, 0.36]	0.21 *	[0.03, 0.39]
3rd quartile	0.41 ***	[0.30, 0.53]	0.35 ***	[0.18, 0.53]
Highest quartile	0.47 ***	[0.33, 0.60]	0.40 ***	[0.19, 0.61]
Unknown	0.04	[−0.16, 0.24]	0.16	[−0.16, 0.48]
Area (ref: urban)				
Rural	−0.12 *	[−0.23, −0.01]	−0.07	[−0.24, 0.10]
Chronic disease (ref: no)				
Yes	0.15 **	[0.04, 0.27]	0.30 **	[0.12, 0.48]
Unknown	−0.08	[−0.38, 0.22]	−0.14	[−0.59, 0.31]
COVID-19 infection of the respondent (ref: no)				
Yes	0.07	[−0.15, 0.30]	0.67 ***	[0.29, 1.06]
Unknown	0.18	[−0.49, 0.84]	1.44 **	[0.40, 2.48]
COVID-19 infection of the respondent’s family members (ref: no)				
Yes	0.05	[−0.14, 0.23]	0.33 *	[0.03, 0.63]
Unknown	0.06	[−0.55, 0.67]	−0.51	[−1.48, 0.47]
Society (ref: Hong Kong)				
Japan	0.47 ***	[0.32, 0.62]	−1.60 ***	[−1.85, −1.34]
Singapore	1.16 ***	[1.01, 1.30]	1.36 ***	[1.16, 1.56]
South Korea	0.90 ***	[0.76, 1.05]	−0.92 ***	[−1.14, −0.71]
UK	1.40 ***	[1.25, 1.56]	2.11 ***	[1.88, 2.35]
US	0.95 ***	[0.79, 1.11]	1.72 ***	[1.49, 1.94]

* *p* < 0.05, ** *p* < 0.01, *** *p* < 0.001. The ^†^ b coefficients were generated using OLS regression. The ^‡^ b coefficients were generated using logistic regression.

**Table 3:** Infodemic, institutional trust, and COVID-19 vaccine hesitancy and uptake.

	Willingness to Accept COVID-19 Vaccines				Uptake of COVID-19 Vaccines			
	Model 1a	Model 2a	Model 3a	Model 4a	Model 1b	Model 2b	Model 3b	Model 4b
IO	0.20 ***	0.12 ***	0.09	0.10 ***	0.13 ***	0.08 **	0.11	0.06 *
	[0.16, 0.23]	[0.09, 0.15]	[−0.02, 0.19]	[0.07, 0.13]	[0.08, 0.18]	[0.02, 0.13]	[-0.08, 0.29]	[0.01, 0.11]
MI	−0.31 ***	−0.24 ***	−0.26 ***	−0.63 ***	−0.20 ***	−0.15 ***	−0.17 ***	−0.47 ***
	[−0.34, −0.29]	[−0.27, −0.21]	[−0.29, −0.23]	[−0.73, −0.53]	[−0.25, −0.15]	[−0.20, −0.10]	[−0.22, −0.12]	[−0.65, −0.28]
Trust in the government		0.25 ***	0.19 ***	0.07 *		0.18 ***	0.01	0.01
		[0.22, 0.28]	[0.11, 0.27]	[0.00, 0.14]		[0.12, 0.24]	[−0.14, 0.16]	[−0.11, 0.14]
Trust in healthcare professionals		0.27 ***	0.33 ***	0.23 ***		0.16 ***	0.45 ***	0.27 **
		[0.23, 0.32]	[0.24,0.43]	[0.14, 0.31]		[0.09, 0.24]	[0.27, 0.64]	[0.10, 0.43]
Trust in NGOs		0.02	−0.06	−0.10 **		0.03	−0.15	−0.21 **
		[−0.01, 0.06]	[−0.15,0.02]	[−0.17, −0.02]		[−0.03, 0.09]	[−0.30, 0.01]	[−0.35, −0.08]
IO × Trust in government			0.02 *				0.05 **	
			[0.00, 0.04]				[0.01, 0.08]	
IO × Trust in healthcare professionals			−0.00				−0.02	
			[−0.02, 0.02]				[−0.15, 0.01]	
IO × Trust in NGOs			0.03 *				0.05 *	
			[0.01, 0.05]				[0.01, 0.08]	
MI × Trust in government				0.06 ***				0.05 **
				[0.04, 0.07]				[0.02, 0.08]
MI × Trust in healthcare professionals				−0.01				−0.05 *
				[−0.03, 0.01]				[−0.09, −0.01]
MI × Trust in NGOs				0.04 ***				0.07 ***
				[0.02, 0.05]				[0.04, 0.10]

Note: All models adjusted for sociodemographic variables, including age, sex, education, occupation, income, rural/urban area, chronic disease, the COVID-19 infection status of the respondents and their family members. OLS regression models were used to assess COVID-19 vaccine hesitancy and logistic regression models were used to assess COVID-19 vaccine uptake 95%, with confidence intervals in brackets. IO = perceived information overload; MI = beliefs in misinformation. * *p* < 0.05, ** *p* < 0.01, *** *p* < 0.001.

## Data Availability

Data cannot be shared publicly because data is in the process of being deposited. Data will be available upon reasonable request from the corresponding author.

## Supplementary Materials

The following supporting information can be downloaded at: https://www.mdpi.com/article/10.3390/ijerph19138033/s1, Table S1. Additional information about sociodemographics of the participants (%); Table S2. Descriptive statistics of key covariates in six jurisdictions; Table S3. Descriptive statistics of independent variables; Supplementary SI. Details of research methodology; Supplementary SII. Survey tool.
