# Supplementary material for: Infodemic, Institutional Trust, and COVID-19 Vaccine Hesitancy: A Cross-National Survey
Source: Int J Environ Res Public Health. 2022 Jun 30;19(13):8033. doi: 10.3390/ijerph19138033 (PMC9265924; doi:10.3390/ijerph19138033)
Supplement: Supplementary file 1 [file ijerph-19-08033-s001.zip › ijerph-1727655-supplementary.pdf]

## Supplementary I. Details of research methodology

### Survey design

The data of this study were obtained from a cross-national online survey of public attitudes and responses toward COVID-19 in six jurisdictions in Asia and Western societies, including Hong Kong, Japan, South Korea, Singapore, the UK, and the US. The survey was conducted between June 15 and June 30, 2021. Reporting adheres to the Checklist for Reporting Results of Internet E-Surveys (CHERRIES).[1]

The surveys were completed using the online panels provided by a global data company. The company's online panels comprise an opt-in list of 56,000 to 1,440,000 individuals relative to the population size in the six jurisdictions surveyed in this study. The survey targeted residents aged 18 or above. A quota sampling strategy was adopted to ensure that the samples chosen match the population's geographical and demographic characteristics released by the latest available census in each jurisdiction. A total number of 6764 representative respondents were collected, with approximately 1100 individuals in each jurisdiction.

### IRB (Institutional Review Board) approval and informed consent process

The study was approved by the Human Subject Ethics Committee of the City University of Hong Kong (Ref No: 8-2020-04-E295-18). All necessary participants consent has been obtained. The informed consent section was shown at the top of the first survey page. They were told the length of the time of the survey, the purpose of the study, and the contact information of the project coordinators. The project team also guaranteed that all the information they provided will be treated with complete confidentiality and anonymity.

Details are as follows.

"This project aims to compare the public health measures, risk perception, and behavior change across six jurisdictions, including four in Asia, one in Europe, and one in North America. The survey will take around 10 minutes to complete. The survey questionnaire is designed by a team of public policy, media communication, and public health experts from the City University of Hong Kong, Hong Kong Baptist University, and The Chinese University of Hong Kong. All the information you provide will be entirely anonymous and confidential. We do not disclose your personal data to any third party without your prior consent. Should you have any questions, please feel free to contact the project coordinator at [email]"

We did not collect the national ID number of the participants. All the information they provided will be entirely anonymous and confidential. We do not disclose their data to any third party without their consent.

For data storage, external hard drives will be used instead of online cloud devices. To ensure drive security, the password will be required for accessing any data. The external drives will be locked at the office to lower the possibility of leakage of data outside the university. The data will be completely erased from all storage devices within 12 months upon the completion of the project.

**Development and pre-testing**
The usability and technical functionality of the electronic questionnaire had been tested by the research team during the pilot stage of this study. The research agency is experienced in developing online survey platforms.

**Recruitment process and description of the sample having access to the questionnaire**
This study adopted a closed/password-protected survey. The data company sends out email invitations to registered panel members, inviting them to log in to their portal to answer surveys in their free time via PC or mobile. The survey was only announced or advertised on the survey platform of the data company when panelists had logged in. The aforementioned informed consent was the announcement of our survey.

**Survey administration**
The survey was conducted between June 15 and June 30, 2021. It required the participants to log in to the platform to answer our survey after receiving email invitations. The questionnaire was available in English, Chinese, Korean, Japanese for participants from different jurisdictions. They received reward points to redeem for cash and prizes upon completing surveys. The data company monitored the completion status of the survey to ensure survey quality and sample representativeness.

The items of some questions, for example, the list of political parties, were randomized to prevent order bias – the bias that generates from the order of answer choices. The platform only displayed one questionnaire item per page, while there were 30 pages for our survey. In addition, it is mandatory for the respondent to answer all the questions by the function of JAVAScript. Accordingly, we did not need to adopt adaptive questioning. All items provided a non-response option, namely Don't know or Not applicable". Participants were also able to review and change their answers via a Back button. The response rates ranged from 15% to 30%, and the overall response rate was 18%.

**Preventing multiple entries from the same individual**
To prevent multiple entries from the same respondent, the survey was never displayed a second

time once the panelist had submitted the survey. IP address check was utilized to prevent the same respondent from answering the survey twice once the whole questionnaire had been submitted. The survey platform measured the time people used to fill in a questionnaire and excluded questionnaires that were submitted too soon (less than three minutes).

**Analysis**

We did not use the weighting of items or propensity scores to adjust for the non-representative sample. Instead, we adopted quota sampling based on the geographic and demographic characteristics obtained from the latest censuses in each of the six cases to ensure balanced representation. Under close monitoring of the project team, Dynata conducted a data checking process to ensure the validity and reliability of the data collected.

**Data handling and analysis**

All data were checked after entry by a trained research assistant on potential errors. Potential errors were then double-checked and corrected if necessary. After data cleaning, we used descriptive statistics to calculate frequencies of prevalence in numbers and their percentages. For normally distributed continuous data, we calculated means and their standard deviations, and for non-normally distributed data, we calculated medians and their interquartile ranges. Next, we used OLS or logistic regression models with robust standard errors to examine the associations between various background variables and two outcome variables—vaccine hesitancy (continuous) and vaccine uptake (binary). We then examined the effects of COVID-19 vaccine-related IO and misinformation on the two outcome variables after adjusting for various sociodemographic variables. Lastly, two-way interaction terms were computed between perceived IO and belief in misinformation about COVID-19 vaccines with each of the four types of institutional trust. If a significant interaction emerged, it showed that the effect of infodemic on vaccine consequences depended on that type of institutional trust. All the analyses were performed using Stata 16.0. Unstandardized coefficients with 95% confidence intervals were reported. A $p$ value of 0.05 was set as the level of statistical significance.

**Role of the funding source**

The funders of the study had no role in study design, data analysis, data interpretation, or writing of the report. All authors had access to all the data in the study and had final responsibility for the decision to submit for publication.

**Reference:**

1. Eysenbach G. Improving the quality of Web surveys: the Checklist for Reporting Results of Internet E-Surveys (CHERRIES). Journal Of Medical Internet Research. 2004 Sep 29;6(3):e132. doi: 10.2196/jmir.6.3.e34

**Supplementary II. Survey tool**

| Variable | Survey question | Coding |
|---|---|---|
| Willingness to accept COVID-19 vaccines | "A few vaccines are developed to prevent coronavirus; would you accept it for yourself?" | 1 = "definitely no"; 7 = "definitely yes" |
| Vaccine uptake | "Have you received the COVID-19 vaccine (at least one jab)" | 1 = "yes"; 0 = "no" |
| Perceived information overload (IO) | Measured by two items adapted from Farooq et al. (2020): (1) "I receive too much information regarding COVID-19 vaccine to form a coherent picture of what is happening in the past four weeks." and (2) "I am often distracted by the excessive amount of information and news on multiple sources about COVID-19 vaccine." | The responses for two items ranged from 1 = "strongly disagree" to 7 = "strongly agree." The average score of the two items was used to indicate perceived IO, with higher values representing higher levels of perceived IO (r = 0.71, p<0.001). |
| Belief in misinformation | Whether participants agreed with three widespread false claims about COVID-19 vaccines: (1) "The real purpose of a mass vaccination program against COVID-19 is to track and control the population." (2) "The COVID-19 vaccine will alter human DNA." and (3) "The only reason the COVID-19 vaccine is being developed is to make money for pharmaceutical companies." | The responses ranged from 1= "strongly disagree" to 7 = "strongly agree." The mean score of the three items was summed up to represent COVID-19 vaccine misinformation, with higher values indicating higher belief in misinformation. The internal reliability of the scale was satisfactory (Cronbach's alpha = 0.89). |
| Trust in the government | How much do you trust (1) your government and (2) public health in handling the COVID-19 outbreak right? | The responses ranged from 1= "totally do not trust" to 7 = "totally trust". The mean score of the two items was calculated to represent trust in the government (r = 0.77, p<0.001) |
| Trust in public health professionals | How much do you trust doctors and nurses in handling the COVID-19 outbreak right? | The responses ranged from 1= "totally do not trust" to 7 = "totally trust". |

| | | |
|---|---|---|
| Trust in NGOs | How much do you trust NGOs in handling the COVID-19 outbreak right? | The responses ranged from 1= “totally do not trust” to 7 = “totally trust” |
| Age | “What is your age group?”<br>Under 20; 20-24; 25-29; 30-34; 35-39; 40-44; 45-49; 50-54; 55-59; 60-64; 65 or above | The age group was collapsed into three groups: 18-29; 30-59; ≥60 |
| Sex | “What is your gender?”<br>Male; Female | 1 = male; 2 = female |
| Education | “What is your education?”<br>Primary or below; Secondary; Tertiary; Graduate school or above; Prefer not to answer | Education variable included three categories: secondary or below (reference group); tertiary education or above; and prefer not to answer |
| Occupation | “What is your occupation?”<br>1. Professional and technical (for example: doctor, teacher, engineer, artist, accountant, nurse); 2. Higher administrative (for example: banker, executive in big business, high government official, union official); 3. Clerical (for example: secretary, clerk, office manager, civil servant, bookkeeper); 4. Sales (for example: sales manager, shop owner, shop assistant, insurance agent, buyer); 5. Service (for example: restaurant owner, police officer, waitress, barber, caretaker); 6. Skilled worker (for example: foreman, motor mechanic, printer, seamstress, tool and die maker, electrician); 7. Semi-skilled worker (for example: bricklayer, bus driver, cannery worker, carpenter, sheet metal worker, baker); 8. Unskilled worker | The responses were collapsed into three groups: professional or service worker (for respondents reporting 1-5; reference group); manual worker (6-10), and other (91-96) |

| | | |
|---|---|---|
| | (for example: laborer, porter, unskilled factory worker, cleaner); 9. Farmworker (for example: farm laborer, tractor driver); 10. Farm proprietor, farm manager; 91. Never had a job; 92. Currently unemployed; 93. Retired; 94. Student; 95. Homemaker; 96. Other | |
| Income | "What is the average monthly income of your household in the past year" | The household income was categorized into quartiles (Q1 (lowest income) to Q4 (highest income). |
| Area | Do you live in an urban or rural area? (Urban; Rural) | 1 = Urban area; 2 = Rural area |
| Chronic disease | "Do you have any chronic illness records?" (Yes; No; Prefer not to answer) | No (reference group); Yes; Prefer not to answer |
| COVID-19 infection of the respondent | Have you been infected with COVID-19? (Yes; No; Prefer not to answer) | No (reference group); Yes; Prefer not to answer |
| COVID-19 infection of the respondent's family members | Have any of your family members been infected with COVID-19? (Yes; No; Prefer not to answer) | No (reference group); Yes; Prefer not to answer |
| Society | There were six societies where the survey was conducted: Hong Kong; Japan; Singapore; South Korea; the UK; and the US. | 1 = Hong Kong; 2 = Japan; 3 = Singapore; 4 = South Korea; 5 = the UK; and 6 = the US. |

**Table S1.** Additional information about sociodemographics of the participants (%)

| | HK | JP | SG | SK | UK | US |
|---|---|---|---|---|---|---|
| Age | | | | | | |
| 18-29 | 23·3 | 20·6 | 22·0 | 23·4 | 21·6 | 23·1 |
| 30-59 | 59·8 | 48·4 | 57·3 | 59·2 | 54·3 | 53·0 |
| ≥60 | 16·9 | 31·3 | 20·7 | 17·4 | 24·1 | 23·9 |
| Sex | | | | | | |
| Male | 48·5 | 51·2 | 49·2 | 50·4 | 47·6 | 47·1 |
| Female | 51·5 | 48·8 | 50·8 | 49·6 | 52·4 | 52·9 |
| Education | | | | | | |
| ≤Secondary | 28·0 | 24·4 | 13·2 | 1·5 | 34·9 | 25·0 |
| ≥Tertiary | 55·1 | 65·2 | 32·9 | 64·9 | 14·1 | 25·2 |
| Unknown/prefer not to answer | 16·9 | 10·4 | 53·9 | 33·6 | 50·9 | 49·8 |
| Occupation | | | | | | |
| Professional/service worker | 84·1 | 57·1 | 78·2 | 63·0 | 50·6 | 50·3 |
| Manual worker | 6·4 | 9·9 | 4·4 | 7·1 | 18·7 | 8·3 |
| Other | 9·5 | 33·0 | 17·3 | 29·9 | 30·7 | 41·5 |
| Income | | | | | | |
| Lowest quartile | 17·1 | 25·1 | 29·7 | 22·6 | 20·5 | 24·3 |
| 2nd quartile | 24·1 | 20·3 | 27·9 | 22·3 | 25·3 | 16·0 |
| 3rd quartile | 39·7 | 21·8 | 23·6 | 29·8 | 26·2 | 21·7 |
| Highest quartile | 11·8 | 14·4 | 10·8 | 16·3 | 13·0 | 17·6 |
| Unknown/prefer not to answer | 7·3 | 18·3 | 8·0 | 9·1 | 15·0 | 20·4 |
| Area | | | | | | |
| Urban | 93·4 | 93·6 | 95·3 | 94·2 | 73·5 | 73·0 |
| Rural | 6·6 | 6·4 | 4·7 | 5·8 | 26·5 | 27·0 |
| Chronic disease | | | | | | |
| No | 82·0 | 73·7 | 81·6 | 76·4 | 75·4 | 65·6 |
| Yes | 10·2 | 16·7 | 11·3 | 16·6 | 14·8 | 19·8 |
| Unknown/prefer not to answer | 7·8 | 9·6 | 7·1 | 7·0 | 9·8 | 14·7 |
| COVID-19 infection of the respondent | | | | | | |
| No | 92·2 | 92·0 | 92·3 | 93·1 | 83·1 | 73·9 |
| Yes | 0·8 | 0·7 | 2·2 | 0·7 | 10·3 | 13·9 |
| Unknown/prefer not to answer | 7·0 | 7·3 | 5·5 | 6·3 | 6·5 | 12·2 |
| COVID-19 infection of the respondent's family members | | | | | | |
| No | 92·3 | 91·7 | 92·4 | 92·9 | 75·2 | 94·9 |
| Yes | 0·9 | 0·9 | 2·2 | 0·7 | 17·9 | 22·8 |
| Unknown/prefer not to answer | 6·7 | 7·4 | 5·4 | 6·4 | 6·9 | 12·3 |

**Table S2.** Descriptive statistics of key covariates in six jurisdictions.

| | HK | | JP | | SG | | SK | | UK | | US | | Group differences* |
|---|---|---|---|---|---|---|---|---|---|---|---|---|---|
| | Mean | SD | Mean | SD | Mean | SD | Mean | SD | Mean | SD | Mean | SD | *p* value |
| Willingness to accept COVID-19 vaccines | 4.75 | 1.84 | 4.75 | 1.84 | 5.88 | 1.41 | 5.71 | 1.52 | 6.11 | 1.50 | 5.70 | 1.92 | $p < 0.000$[1] |
| Uptake of COVID-19 vaccines (1 = yes) | 0.34 | 0.47 | 0.34 | 0.47 | 0.67 | 0.47 | 0.19 | 0.39 | 0.81 | 0.39 | 0.76 | 0.43 | $p < 0.000$[2] |
| Perceived information overload | 4.03 | 1.28 | 4.03 | 1.28 | 4.36 | 1.39 | 4.31 | 1.37 | 4.10 | 1.66 | 4.18 | 1.78 | $p < 0.000$[3] |
| Belief in misinformation | 3.91 | 1.44 | 3.91 | 1.44 | 3.89 | 1.54 | 3.44 | 1.63 | 3.32 | 1.94 | 3.77 | 2.04 | $p < 0.000$[4] |
| Trust in the government | 4.14 | 1.61 | 3.46 | 1.56 | 5.56 | 1.19 | 5.05 | 1.45 | 4.69 | 1.44 | 4.70 | 1.62 | $p < 0.000$[5] |
| Trust in public health professionals | 5.07 | 1.21 | 4.96 | 1.30 | 5.72 | 1.07 | 5.29 | 1.12 | 5.48 | 1.40 | 5.30 | 1.51 | $p < 0.000$[6] |
| Trust in local NGOs | 4.43 | 1.10 | 4.43 | 1.10 | 4.93 | 1.18 | 4.48 | 1.27 | 4.58 | 1.41 | 4.56 | 1.58 | $p < 0.000$[7] |

Note: * the group differences were calculated by one-way ANOVA.

[1]Further two independent sample *t*-test between jurisdictions showed no significant differences between UK and US; comparisons between any other two jurisdictions were significant.

[2]Further two independent sample *t*-test between jurisdictions showed no significant differences between HK and JP, HK and US, JP and UK, JP and US, SG and SK, SG and US. SK and US, UK and US; comparisons between any other two jurisdictions were significant.

[3]Further two independent sample *t*-test between jurisdictions showed no significant differences between HK and SG, HK and US, JP and SK, JP and US, SG and US, SK and UK; comparisons between any other two jurisdictions were significant.

[4]Further two independent sample *t*-test between jurisdictions showed no significant differences between UK and US; comparisons between any other two jurisdictions were significant.

[5]Further two independent sample *t*-test between jurisdictions showed no significant differences between SK and UK, SK and US; comparisons between any other two jurisdictions were significant.

[6]Further two independent sample *t*-test between jurisdictions showed no significant differences between SG and UK, UK and US; comparisons between any other two jurisdictions were significant.

[7]Further two independent sample *t*-test between jurisdictions showed no significant differences between HK and SK, HK and UK, HK; comparisons between any other two jurisdictions were significant. and US, SK and UK, SK and US, UK and US

**Table S3.** Descriptive statistics of independent variables.

| Variable | % | Variable | % |
|---|---|---|---|
| I receive too much information regarding COVID-19 vaccine to form a coherent picture of what is happening in the past four weeks. | | The only reason the COVID-19 vaccine is being developed is to make money for pharmaceutical companies. | |
| Strongly disagree | 7.3 | Strongly disagree | 10.7 |
| Disagree | 10.3 | Disagree | 7.6 |
| Somewhat disagree | 13.8 | Somewhat disagree | 11.5 |
| Neither agree nor disagree | 27.1 | Neither agree nor disagree | 17.2 |
| Somewhat agree | 20.1 | Somewhat agree | 19.5 |
| Agree | 14.2 | Agree | 22.0 |
| Strongly agree | 7.19 | Strongly agree | 11.6 |
| I am often distracted by the excessive amount of information and news on multiple sources about COVID-19 vaccine. | | Trust in the government | |
| Strongly disagree | 6.9 | Totally do not trust | 10.7 |
| Disagree | 9.5 | Do not trust | 7.6 |
| Somewhat disagree | 13.0 | Somewhat do not trust | 11.5 |
| Neither agree nor disagree | 24.4 | Neither trust nor distrust | 17.2 |
| Somewhat agree | 23.9 | Somewhat trust | 19.5 |
| Agree | 15.6 | Trust | 22.0 |
| Strongly agree | 6.6 | Totally trust | 11.6 |
| The real purpose of a mass vaccination program against COVID-19 is to track and control the population. | | Trust in healthcare professionals | |
| Strongly disagree | 20.1 | Totally do not trust | 5.2 |
| Disagree | 13.2 | Do not trust | 5.5 |
| Somewhat disagree | 10.6 | Somewhat do not trust | 8.6 |
| Neither agree nor disagree | 20.2 | Neither trust nor distrust | 17.4 |
| Somewhat agree | 14.6 | Somewhat trust | 22.2 |
| Agree | 13.5 | Trust | 27.5 |
| Strongly agree | 7.9 | Totally trust | 13.6 |
| The COVID-19 vaccine will alter human DNA. | | Trust in NGOs | |
| Strongly disagree | 20.3 | Totally do not trust | 3.6 |
| Disagree | 13.1 | Do not trust | 4.1 |
| Somewhat disagree | 10.0 | Somewhat do not trust | 8.3 |
| Neither agree nor disagree | 23.5 | Neither trust nor distrust | 35.5 |
| Somewhat agree | 14.6 | Somewhat trust | 26.0 |
| Agree | 11.8 | Trust | 16.2 |
| Strongly agree | 6.8 | Totally trust | 6.3 |
